# Cancer Risk and Mortality Following Kaposi Sarcoma Among People with HIV in the United States, 2000 to 2019

**DOI:** 10.1007/s10552-025-02105-0

**Published:** 2026-01-17

**Authors:** Sally Peprah, Eric A. Engels, Qianlai Luo, Analise Monterosso, Colby H. Cohen, Ruth M. Pfeiffer, Meredith S. Shiels

**Affiliations:** 1 Division of Cancer Epidemiology and Genetics, National Cancer Institute, 9609 Medical Center Drive, RM 6-E218, Bethesda, MD 20892 USA; 2 Texas Department of State Health Services, Austin, TX USA; 3 Florida Department of Health, Tallahassee, FL USA

**Keywords:** HIV, Kaposi sarcoma, Survival

## Abstract

People with HIV (PWH) have increased Kaposi sarcoma (KS) risk. Among 2,645 PWH with KS in the United States HIV/AIDS Cancer Match Study, subsequent cancer risk was elevated 2.8-fold (95% confidence interval [CI]: 2.4–3.3) compared to PWH without KS. Mortality after KS declined 2000–2002 and 2012–2015 (hazard ratio = 0.64; 95%CI: 0.55–0.74).

**Supplementary Information:**

The online version contains supplementary material available at 10.1007/s10552-025-02105-0.

## Introduction

Kaposi sarcoma (KS) is caused by KS herpesvirus (KSHV) and is associated with immunosuppression in people with HIV (PWH) [1]. With widespread access to improved combination antiretroviral therapy (cART), life expectancy has increased, and KS rates have declined, though they remain elevated relative to the general population [2]. Survival after KS among PWH has also improved over time; for example, 2-year survival in men who have sex with men (MSM) increased from 35% in 1986–1995 to 81% in 2000–2006 [3]; nonetheless, disparities in KS survival among PWH have been reported [4]. Therefore, more recent estimates of KS mortality among key demographic and HIV transmission risk factors and by calendar period are needed.

Due to shared etiologic factors and immunosuppression, PWH have an increased overall risk of developing multiple cancers [5]; however, data on cancer risk following KS are limited. Previous studies including studies in the pre-cART era [1, 6] suggest an elevated cancer risk following KS, particularly for lymphohematopoietic malignancies [1, 6, 7]. However, more recent studies had limited size, length of follow-up or utilized the general population as comparator [5, 7]. Thus, using population-level linked HIV and cancer registry data, we evaluated subsequent cancer risk and mortality after KS in U.S. PWH.

## Methods

Data from the HIV/AIDS Cancer Match (HACM) Study, a linkage of HIV and cancer registries across 12 U.S. regions (Supplementary Table 1), were utilized. Namely, data from the following regions: Colorado (2000–2015), Michigan (2000–2015), New Jersey (2000–2012), Texas (2000–2015), Louisiana (2000–2015), New York (2001–2019), Puerto Rico (2003–2017), Georgia (2004–2012), Connecticut (2000–2015), Washington DC (2007–2015), Maryland (2008–2018) and North Carolina (2000–2014) were included for the years indicated. Follow-up began at the latter of the start of registry coverage, HIV report date, and age 15 years, and ended at the earliest of non-KS cancer diagnosis (outcome), death, end of registry coverage or December 31, 2019. Only PWH with no cancer history prior to follow-up start were included.

The outcome of second cancer after KS was defined as the risk of a non-KS cancer diagnosis following a primary KS diagnosis (minimum time between KS diagnosis and second cancer diagnosis was one month). The relative risk of second cancer after KS was estimated using adjusted hazard ratios [aHRs] and 95% confidence intervals [CIs] from Cox regression models with age as timescale, non-KS cancer diagnosis as the outcome and primary KS diagnosis as a time-varying exposure. Adjustments were made using age at the start of follow-up for 5-year age groups (15–29, 30–39, 40–49, 50–59 and ≥ 60), HIV transmission risk factor (MSM including MSM/people who inject drugs [PWID], males and females PWID excluding MSM/PWID, male heterosexual/other and female heterosexual/other), race/ethnicity (White, Black, Hispanic and other/unknown), region, and year of HIV diagnosis. This model was also run separately with outcomes restricted to the four most common cancers observed (non-Hodgkin lymphoma (NHL), and anal, prostate and lung cancers). A sensitivity analysis was restricted to people with an AIDS diagnosis at baseline, i.e., PWH with notable immune suppression.

Separate Cox regression models were used to assess mortality risk following primary KS and to examine temporal changes in survival. These models were stratified by 5-year baseline age groups (defined above) with time since KS diagnosis as timescale, adjusted for region of enrollment, baseline age on a continuous scale, HIV transmission risk factor, region, race/ethnicity and HIV diagnosis year. Follow-up ended at the earliest of death, end of registry coverage or December 31, 2019. Estimates of 5-year probability of death were obtained as output tables from the adjusted Cox regression model. Statistical analyses were conducted in SAS 9.4.

## Results

There were 605,566 PWH with 5,063,484 person-years of follow-up, including 2,645 incident KS cases (22,556 person-years). Mean age of patients at KS diagnosis was 36.5 years (standard deviation = 9.4). Individuals with KS were predominantly Black (42.8%, n = 1,132; Table 1), and MSM (73.2%, n = 1,936). Two-thirds of KS diagnosis occurred during 2000–2005 (67.0%, n = 1229), 16.6% (n = 439) in 2006–2008 and 16.5% (n = 435) in 2009–2019.

**Table 1:** Adjusted hazard ratios of death and 5-year probability of death after primary KS diagnosis in the HACM study, 2000–2019

	Overall PWH without cancer diagnosis at start of follow up (*N* = 605,566)	PWH diagnosed with KS during follow-up (*N* = 2,645)	Hazard ratio* (95%CI)	Adjusted hazard ratio*† (95%CI)	5-year probability of death, %
	*n* (%)				
Race/Ethnicity					
White	136,445 (22.5)	623 (23.6)	Ref	Ref	32.1 (29.8, 34.6)
Black	270,476 (44.7)	1,132 (42.8)	1.63 (1.48, 1.81)	1.56 (1.40, 1.74)	48.8 (46.5, 51.2)
Hispanic	150,330 (24.8)	722 (27.3)	1.08 (0.97, 1.21)	1.01 (0.89, 1.14)	34.4 (32.0, 37.0)
Other	30,438 (5.0)	143 (5.4)	0.88 (0.72, 1.07)	0.94 (0.77, 1.14)	31.9 (27.2, 37.3)
Unknown§	17,877 (3.0)	25 (1.0)	–	–	–
HIV transmission risk factor					
Men who have sex with men	268,704 (44.4)	1,936 (73.2)	Ref	Ref	34.7 (33.2, 36.3)
Males and females who inject drugs	93,914 (15.5)	240 (9.1)	2.22 (1.96, 2.51)	2.06 (1.80, 2.34)	63.1 (58.5, 67.7)
Male heterosexual/other	116,991 (19.3)	364 (13.8)	1.27 (1.14, 1.42)	1.20 (1.07, 1.35)	42.4 (39.0, 45.9)
Feale heterosexual/other	125,957 (20.8)	105 (4.0)	1.59 (1.28, 1.96)	1.45 (1.16, 1.80)	49.9 (42.3, 58.1)
Year of KS diagnosis					
2000–2002	189,827 (31.4)	1,246 (47.1)	Ref	Ref	43.1 (40.6, 45.7)
2003–2005	99,426 (16.4)	525 (19.9)	1.02 (0.91, 1.14)	0.94 (0.84, 1.06)	45.9 (42.6, 49.2)
2006–2008	104,814 (17.3)	439 (16.6)	0.76 (0.67, 0.85)	0.76 (0.67, 0.86)	36.2 (33.4, 39.2)
2009–2011	88,665 (14.6)	258 (9.8)	0.70 (0.62, 0.80)	0.70 (0.61, 0.80)	34.0 (31.1, 37.2)
2012–2015	92,475 (15.3)	149 (5.6)	0.64 (0.56, 0.74)	0.64 (0.55, 0.74)	29.5 (26.4, 32.9)
p-trend			< 0.0001	< 0.0001	
2016–2019¶	30,359 (5.0)	28 (1.1)	–	–	–

^*^ Baseline hazard stratified by 5-year age groups (15–29, 30–39, 40–49, 50–59 and ≥ 60) and model adjusted for age on a continuous scale

^**†**^ Baseline hazard stratified by 5-year age groups and model further adjusted for age on a continuous scale, HIV transmission risk factor, region, race/ethnicity and year of KS diagnosis

^§^ Data for unknown category of race/ethnicity not included in the Cox model

^¶^ This category of calendar year of KS diagnosis was not included in the Cox model because of follow-up was less than 5-years and as such corresponding estimates could not be obtained. Data from: Colorado (2000–2015), Michigan (2000–2015), New Jersey (2000–2012), Texas (2000–2015), Louisiana (2000–2015), New York (2001–2019), Puerto Rico (2003–2017), Georgia (2004–2012), Connecticut (2000–2015), Washington DC (2007–2015), Maryland (2008–2018) and North Carolina (2000–2014) were included

Cancers following primary KS were diagnosed in 161 participants (37.0% NHL, 7.5% anal cancer, 6.2% prostate cancer, 5.0% lung cancer and 44.3% other cancers; Supplementary Table 2), and risk of cancer following primary KS for PWH was 2.82 times (95%CI: 2.37–3.33; Supplementary Table 3) that of PWH without KS. This risk was further elevated (aHR = 4.25; 95%CI: 3.53–5.05; Supplementary Table 3) when the analysis was restricted to people with AIDS. NHL risk following KS was increased (aHR = 5.06; 95%CI: 3.85–6.51) in PWH and remained elevated when restricted to people with AIDS (aHR = 4.90; 95%CI: 3.62–6.46). Anal cancer risk was also elevated overall (aHR = 2.20; 95%CI: 1.14–3.77) and among people with AIDS (aHR = 2.69; 95%CI: 1.28–4.85). Likewise, lung cancer risk was elevated overall (aHR = 2.33; 95%CI: 1.06–4.34) and in people with AIDS (aHR = 4.07; 95%CI: 1.61–8.28). Among men, prostate cancer risk was not elevated overall (aHR = 1.69; 95%CI: 0.73–3.28) but was increased in men with AIDS (aHR = 2.58; 95%CI: 1.02–5.24; Supplementary Table 3).

Black PWH with KS (5-year mortality: 48.8%; aHR = 1.56, 95%CI: 1.40–1.74; Table 1) had significantly higher mortality compared to White PWH with KS (5-year mortality: 32.1%). However, mortality after KS was not significantly different for Hispanic PWH or other racial/ethnic groups compared to White PWH. MSM had the lowest 5-year mortality following KS (34.7%), with an increased 5-year mortality observed among PWID (63.1%; aHR = 2.06, 95%CI: 1.80–2.34, compared to MSM), and in both men (5-year mortality: 42.4%; aHR = 1.20, 95%CI: 1.07–1.35) and women (5-year mortality: 49.9%; aHR = 1.45, 95%CI: 1.16–1.80) with heterosexual contact or other HIV transmission risk factors. Beginning in 2006–2008 there was a significant decline in mortality following KS across calendar periods (p-trend < 0.0001). Compared to PWH diagnosed with KS in 2000–2002, mortality was lower in 2006–2008 (aHR = 0.76; 95%CI: 0.67–0.86), 2009–2011 (aHR = 0.70; 95%CI: 0.61–0.80) and 2012–2015 (aHR = 0.64; 95%CI: 0.55–0.74). The 5-year mortality rate was 43.1% among people with KS diagnosed in 2000–2002, 45.9% in 2003–2005, 36.2% in 2006–2008, 34.0% in 2009–2010 and 29.5% in 2012–2015.

## Discussion

Among PWH, KS was associated with increased cancer risk, and this increase was more pronounced among people with AIDS. Risks of NHL, anal cancer and lung cancer were elevated among PWH with KS compared to PWH without KS. Mortality following KS in PWH decreased significantly over time; however, Black PWH and PWID had a higher risk of death following KS.

The overall increased risk of cancer following KS is consistent with the literature [4, 7] and pre-cART era studies [1, 6] that have also demonstrated a similar positive association particularly for lymphohematopoietic malignancies [1, 6, 7]. However, the more recent studies have been limited geographically [5] or estimated the prevalence and not incidence of second cancers [8]. The increased risk of cancer following KS may reflect HIV-associated immunosuppression, which is linked with uncontrolled HIV and low CD4 counts. Immunosuppression strongly increases the risk of KS, as well as NHL and anal cancer; thus HIV-induced suppression of the immune system likely contributes to the elevated rate of these cancers after KS. These associations remained even in analyses restricted to people with AIDS, who all (both those with and without KS) have been profoundly immunosuppressed at some point, and immunosuppression may also contribute to the increased risk of lung cancer after KS [9]. The increased risk of NHL among PWH with and without AIDS may also be explained by the subset of immunoblastic lymphomas in people with AIDS-associated KS that have been immunohistochemically linked to KSHV [10].

The decline in KS mortality beginning in 2006–2008 corresponds to improvements in early initiation of cART and in the treatment and care of PWH. Despite these improvements, KS mortality remains significantly elevated among Black PWH, which is consistent with previous work showing poorer KS survival in Black compared to White PWH [4]. Racial differences in mortality following KS may partly be attributed to differences in linkage to and engagement in care, or a later stage of presentation at a health facility with more severe disease for Black PWH [11]. However, these studies were based on limited geographic areas of the U.S., examined survival over shorter or earlier calendar periods in the current treatment era and compared risk in people presumed to have HIV with the general population.

Although MSM have a well-known increased KS risk [2], a relatively lower mortality risk and 5-year probability of death compared to the other HIV transmission risk factors examined was noted. This may be due to increased clinical surveillance or better access to KS treatment for MSM compared to other PWH resulting in earlier detection and better prognosis. In contrast, the poorer survival among PWID could be driven by delayed HIV and KS treatment, due to inadequate linkage to care [12].

This study has several strengths including the use of large population-linked U.S. cancer and HIV registry data. Additionally, this study provides a contemporary examination of mortality risk and of subsequent cancers among PWH with KS in the post-cART era. Our findings are limited by the differential number of years contributed by each registry (see Table 1 note); however, the results were consistent when each registry was systematically excluded, indicating that the observed associations were not solely driven by a particular registry or calendar period of follow-up. In addition, there were a limited number of cancer cases following KS diagnoses, and data on CD4 cell counts, HIV treatment status and other risk factors including smoking and family history of cancer were not available.

These findings demonstrate increased cancer risk following KS in PWH and that this risk is further elevated in those diagnosed with AIDS. Although there has been a significant decline in mortality following KS in recent years, disparities by race/ethnicity and HIV transmission risk factor persist. Thus, continued expansion of access to HIV testing with early initiation of cART, in addition to ensuring cancer screening and treatment in PWH are consistent with guidelines, may be important for potentially mitigating cancer risk and mortality following KS.

## Supplementary Information

Below is the link to the electronic supplementary material.

Supplementary file1 (DOCX 16 kb)

## Data Availability

No datasets were generated or analysed during the current study.

## Declarations

### Disclaimer

The views expressed in this paper are those of the authors and should not be interpreted to reflect the views or official policies of the NCI, HIV/AIDS or cancer registries, the U.S. FDA, the Centers for Disease Control and Prevention, or their contractors, nor does the mention of trade names, commercial practices, or organizations imply endorsement by the U.S. Government.
