# Supplementary material for: Cancer Risk and Mortality Following Kaposi Sarcoma Among People with HIV in the United States, 2000 to 2019
Source: Cancer Causes Control. 2026 Jan 17;37(2):23. doi: 10.1007/s10552-025-02105-0 (PMC12812070; doi:10.1007/s10552-025-02105-0)
Supplement: Supplementary file 1 — Supplementary file1 (DOCX 16 kb) [file 10552_2025_2105_MOESM1_ESM.docx]

**Supplementary Table 1.** Regional distribution of participants with a primary KS diagnosis in the HACM study with corresponding person-time contribution, 2000–2015.

| **Region** | **Years** | **Overall** | | **Diagnosed with primary KS during follow-up** | |
| --- | --- | --- | --- | --- | --- |
|  |  | **n** | **Person-years** | **n** | **Person-years** |
| Colorado | 2000-2015 | 17,513 | 171,804 | 109 | 1,148 |
| Connecticut | 2002-2016 | 13,377 | 123,881 | 32 | 287 |
| Washington DC | 2007-2015 | 20,288 | 123,119 | 36 | 223 |
| Georgia | 2004-2012 | 57,428 | 264,543 | 205 | 930 |
| Louisiana | 2000-2015 | 33,016 | 262,956 | 148 | 1,217 |
| Maryland | 2008-2018 | 30,396 | 211,954 | 63 | 481 |
| Michigan | 2000-2015 | 22,609 | 198,238 | 111 | 980 |
| North Carolina | 2000-2014 | 42,592 | 324,248 | 112 | 849 |
| New Jersey | 2000-2012 | 45,118 | 365,735 | 149 | 1,049 |
| New York | 2001-2019 | 185,694 | 1,912,536 | 887 | 8,942 |
| Puerto Rico | 2003-2017 | 26,590 | 232,760 | 84 | 585 |
| Texas | 2000-2015 | 110,945 | 871,710 | 709 | 5,864 |
| **Total** | **2000-2019** | **605,566** | **5,063,484** | **2,645** | **22,555** |

**Supplementary Table 2.** Types of second cancers recorded after a primary KS diagnosis in the HACM study, 2000–2019.

| **Second cancer type** | **n (%)** |
| --- | --- |
| Oral Cavity and Pharynx | 7 (4.4) |
| Stomach | 3 (1.9) |
| Colon Excluding Rectum | 3 (1.9) |
| Rectum and Rectosigmoid Junction | 2 (1.2) |
| Anus, Anal Canal and Anorectum | 12 (7.5) |
| Liver | 6 (3.7) |
| Pancreas | 1 (0.6) |
| Larynx | 1 (0.6) |
| Lung and Bronchus | 8 (5.0) |
| Soft Tissue including Heart | 1 (0.6) |
| Melanoma of the Skin | 4 (2.5) |
| Breast | 1 (0.6) |
| Cervix Uteri | 2 (1.2) |
| Prostate | 10 (6.2) |
| Testis | 3 (1.9) |
| Penis | 3 (1.9) |
| Kidney and Renal Pelvis | 3 (1.9) |
| Brain | 3 (1.9) |
| Thyroid | 1 (0.6) |
| Hodgkin Lymphoma | 7 (4.4) |
| Non-Hodgkin Lymphoma | 61 (37.9) |
| Myeloma | 1 (0.6) |
| Myeloid and Monocytic Leukemia | 4 (2.5) |
| Poorly specified histology at any site | 4 (2.5) |
| Miscellaneous | 10 (6.2) |
| No Cancer | 2,862 |

**Supplementary Table 3.** Hazard ratios of second cancer risk after a primary KS diagnosis in the HACM study, 2000-2019. The risk is shown in columns for both the overall and the restricted analysis starting at AIDS diagnosis date and beyond, row results show risk for all cancer types and the most common cancer types observed.

|  | **Overall** | | **AIDS diagnosed** | |
| --- | --- | --- | --- | --- |
| **Second cancer type** | **Hazard ratio**  **(95% CI)** | **Adjusted hazard ratio**  **(95% CI) *** | **Hazard ratio**  **(95% CI)** | **Adjusted hazard ratio**  **(95% CI) *** |
| All cancers | 2.95 (2.48, 3.48) | 2.82 (2.37, 3.33) | 4.12 (3.42, 4.89) | 4.25 (3.53, 5.05) |
| Non-Hodgkin lymphoma | 5.32 (4.04, 6.83) | 5.06 (3.85, 6.51) | 5.07 (3.75, 6.68) | 4.90 (3.62, 6.46) |
| Anal cancer | 2.88 (1.50, 4.95) | 2.20 (1.14, 3.77) | 3.44 (1.65, 6.21) | 2.69 (1.28, 4.85) |
| Lung cancer | 2.21 (1.01, 4.11) | 2.33 (1.06, 4.34) | 3.93 (1.56, 7.99) | 4.07 (1.61, 8.28) |
| Prostate cancer† | 1.49 (0.64, 2.88) | 1.69 (0.73, 3.28) | 2.20 (0.87, 4.45) | 2.58 (1.02, 5.24) |

* Model adjusted for 5-year age groups, HIV transmission risk factor, region, race/ethnicity and year of diagnosis.

**†** Model restricted to males only.
